# Modeling the Historical and Future Potential Global Distribution of the Pepper Weevil *Anthonomus eugenii* Using the Ensemble Approach

**DOI:** 10.3390/insects16080803

**Published:** 2025-08-03

**Authors:** Kaitong Xiao, Lei Ling, Ruixiong Deng, Beibei Huang, Qiang Wu, Yu Cao, Hang Ning, Hui Chen

**Affiliations:** 1 Hubei Key Laboratory of Biological Resources Protection and Utilization, Hubei Minzu University, Enshi 445000, China; 13402709191@163.com (K.X.); marsyu0722@163.com (R.D.); hbei0825@163.com (B.H.); lxwq20030226@163.com (Q.W.); zhedaotinihui@163.com (Y.C.); 2 College of Forestry and Horticulture, Hubei Minzu University, Enshi 445000, China; 3 College of Biology, Hunan University, Changsha 410082, China; linglei@hnu.edu.cn; 4 State Key Laboratory for Conservation and Utilization of Subtropical Agro-Bioresources, Guangdong Key Laboratory for Innovative Development and Utilization of Forest Plant Germplasm, College of Forestry and Landscape Architecture, South China Agricultural University, Guangzhou 510642, China

**Keywords:** *Anthonomus eugenii*, climate change, ensemble model, potential distribution, pest management

## Abstract

The pepper weevil *Anthonomus eugenii* is a devastating pest native to Central America that can cause severe damage to over 35 pepper varieties. Global trade in peppers has significantly increased the risk of its spread and expansion. Moreover, future climate change may add more uncertainty to its distribution, resulting in considerable ecological and economic damage globally. Therefore, we employed an ensemble model combining Random Forests and CLIMEX to predict the potential global distribution of *A. eugenii* in historical and future climate scenarios. The results indicated that the maximum temperature of the warmest month is an important variable affecting global *A. eugenii* distribution. Under the historical climate scenario, the potential global distribution of *A. eugenii* is concentrated in the Midwestern and Southern United States, Central America, the La Plata Plain, parts of the Brazilian Plateau, the Mediterranean and Black Sea coasts, sub-Saharan Africa, Northern and Southern China, Southern India, Indochina Peninsula, and coastal area in Eastern Australia. Under future climate scenarios, suitable areas in the Northern Hemisphere, including North America, Europe, and China, are projected to expand toward higher latitudes. In China, the number of highly suitable areas is expected to increase significantly, mainly in the south and north. Contrastingly, suitable areas in Central America, northern South America, the Brazilian Plateau, India, and the Indochina Peninsula will become less suitable. The total land area suitable for *A. eugenii* under historical and future low- and high-emission climate scenarios accounted for 73.12, 66.82, and 75.97% of the global land area (except for Antarctica), respectively. The high-suitability areas identified by both models decreased by 19.05 and 35.02% under low- and high-emission scenarios, respectively. Building on these findings, we inferred the future expansion trends of *A. eugenii* globally. Furthermore, we provide early warning of *A. eugenii* invasion and a scientific basis for its spread and outbreak, facilitating the development of effective quarantine and control measures.

## Simple Summary

The pepper weevil is a destructive insect pest from Central America that damages over 35 pepper varieties. Its spread is increasing due to the global pepper trade, and future climate change could make the problem more severe, causing significant harm to crops worldwide. To understand where this pest could survive now and in the future, this study used the ensemble model (constructed using CLIMEX and Random Forests models) combining pest physiology and climate data. The result shows that hot summer temperatures strongly influence where the weevil can survive. Currently, high-risk areas include the southern US, Central and South America, parts of Africa, Southern Europe, Southern and Eastern Asia, and coastal Australia. Under future climate conditions, the areas suitable for the weevil are expected to expand northwards in North America, Europe, and China, potentially increasing damage in these regions, especially in China. However, suitability may decrease in some current hotspots like Central America, northern South America, India, and Southeast Asia. Overall, a very large portion of the world’s land (67–76%) remains suitable for this pest under changing climates. This study helps predict where the pepper weevil might invade next, providing crucial early warnings to improve control of this pest globally.

## 1. Introduction

The pepper weevil *Anthonomus eugenii* Cano is a devastating pest native to Central America that seriously harms commercial cultivars of more than 35 pepper types, with yield losses of 33–90% [1]. The weevil was first discovered in Mexico in 1907 and spread to North and Central America, as well as the Caribbean, and then northward to Texas in 1924, where it is now distributed throughout the southern states of the USA [2]. *A. eugenii* had already spread to Canada in 1993 and invaded Southern Europe in 2014 [3,4]. As a globally produced crop, pepper has experienced accelerated spread and expansion of pepper weevils due to frequent global trade and international transportation [5].

The primary hosts of *A. eugenii* include cultivated *Capsicum* spp., including *C. annuum* and *C. frutescens*, as well as some wild *Capsicum* spp. [6,7]. *A. eugenii* typically infests peppers during the bud and young fruit stages, primarily sucking sap from the buds, flowers, and young fruits [8]. This gradually results in the shedding of large amounts of these organs. During the bud stage, *A. eugenii* survives by feeding on stamens and pollen due to the lack of fruit [9]. When damage is severe, 70–90% of buds are destroyed. During the young fruit stage, *A. eugenii* inflicts the most severe damage [10]. Both larvae and adults feed on young fruits, leading to premature fruit drop. Fruit drop is the most common and obvious characteristic of the damage caused by *A. eugenii* [11]. Approximately 500 pepper weevils can decimate 1 hm^2^ of pepper plants in heavily infested areas, causing substantial economic and ecological losses [12].

Global warming is undeniable. According to the Intergovernmental Panel on Climate Change (IPPC) Fifth Assessment Report (AR5), global temperatures are projected to rise between 0.3 and 4.8 °C by the end of the 21st century [13]. As poikilothermic organisms, insects are significantly affected by changes in global temperature, which influence their growth and development rates, population dynamics, and geographical distribution [14,15]. Recent studies have shown that climate change has altered the geographical distributions of several insects. Insect distribution is expected to expand to high-latitude regions, the two poles, or new regions [16,17,18]. Depending on their climatic tolerance limits, some species may adapt to the new climate in their expanded habitats, whereas others may migrate or become extinct [19].

Identifying the geographical distribution suitable for pests under climate change scenarios is essential for developing long-term management strategies. Species distribution models (SDMs), including BIOCLIM, DOMAIN, GARP, Random Forests (RF), MaxEnt, and CLIMEX, are widely used to project the potential geographical distributions of species [20,21,22]. These models are generally categorized into two types: mechanistic and correlative [23]. Correlative models leverage the statistical relationship between species occurrence and environmental spatial data, operating under the assumption that the current distribution accurately reflects the species’ ecological requirements [24]. Currently, MaxEnt and RF models, developed based on machine learning algorithms, are being used by scholars to project the potential distribution range of species [25]. CLIMEX, a typical mechanistic model that considers the physiological constraints of a species’ habitat, is broadly applied to predict the potential distribution of pests [26]. However, many studies have reported limitations in predictions using a single SDM [27,28]. Therefore, ensemble models combining multiple SDMs are increasingly preferred, significantly improving prediction accuracy. Notwithstanding, several researchers still prefer single SDMs. To better leverage the advantages of mechanistic and correlative SDMs (reciprocal modeling) and to more realistically and effectively reflect the interpretability and transferability of the potential geographical distribution of species, an ensemble model combining two types of SDMs is a priority in species distribution prediction [29].

Research on *A. eugenii* is currently focused on its biology, damage characteristics, host preference, and preventive and control measures [9]. However, a comprehensive understanding of how climate change may influence the distribution of *A. eugenii* globally is lacking. Therefore, an ensemble model based on RF and CLIMEX models was used to project the potential global distribution of *A. eugenii* under historical and future climate change scenarios. This research had four objectives: (1) to explore the potential global distribution of *A. eugenii* under the current climate scenario and determine the key climatic factors affecting its distribution; (2) to predict its potential global distribution under future climate scenarios; (3) to estimate possible expansion or contraction trends of the species’ range with global warming; and (4) to obtain the necessary theoretical basis for monitoring, early warning, and effective prevention and control of the species, which in turn provides an important reference for countries to control the spread of *A. eugenii*.

## 2. Materials and Methods

### 2.1. Overall Modeling Workflow

First, the RF was used to project the potential global distribution of *A. eugenii*, *C. annuum*, and *C. frutescens*. Second, CLIMEX 4.0.2 (Hearne Scientific Software Company, Melbourne, VIC, Australia) was used to project the potential global distribution of *A. eugenii* [26]. Third, the potential global area where *C. annuum* or *C. frutescens* is distributed was determined as the potential global host distribution for *A. eugenii*. Fourth, we used the global host potential distribution as a mask to extract the global potential distribution of *A. eugenii* predicted using RF and CLIMEX. Fifth, we integrated the two results obtained in the fourth step to represent the potential global distribution of *A. eugenii* predicted using the ensemble model.

### 2.2. Acquisition of Global Presence Records of A. eugenii, C. annuum, and C. frutescens

**Figure 1:**
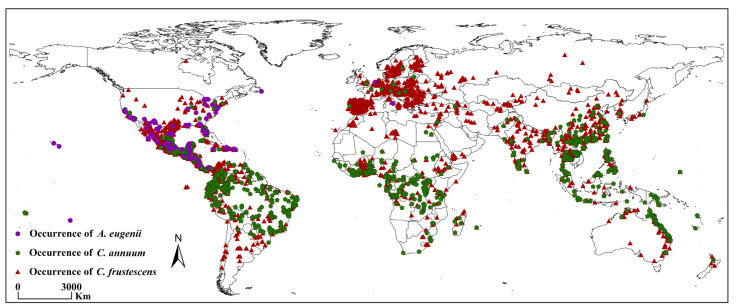
Occurrence records of *A. eugenii*, *C. annuum*, and *C. frutescens*.

Current global occurrence records of *A. eugenii* were collected from the Global Biodiversity Information Facility (GBIF, https://www.gbif.org/, accessed on 20 May 2024), European and Mediterranean Plant Protection Organization (EPPO, https://gd.eppo.int, accessed on 20 May 2024), and Centre for Agriculture and Bioscience International (CABI, https://www.cabi.org/, accessed on 21 May 2024) [30]. Related literature was obtained from the Web of Science (https://clarivate.com.cn, accessed on 21 May 2024) [9]. Subsequently, these datasets were merged, and the “Spatially Rarefy Occurrence Data” function within the SDM toolbox of ArcGIS 10.8 (Environmental System Research Institute Inc., Redlands, CA, USA) was utilized to filter the data. The buffer radius was set to 10 km, and 92 coordinates were obtained for *A. eugenii* (Table S1) [27]. The current global presence records of the host plants *C. frutescens* and *C. annuum* were obtained similarly from the GBIF, EPPO, and CABI databases (Tables S2 and S3) [31,32]. The global presence records of *A. eugenii* and its hosts are shown in Figure 1.

### 2.3. Selection of Climate Data in Different Climate Scenarios

CLIMEX uses fixed-format Location (.loc) and Meteorology (.met) files to store a station’s geographic and climate data, which are then combined into MetManager (.mm) files for use in the software [26]. The .met files contained data for the daily minimum temperature (Tmin), daily maximum temperature (Tmax), monthly precipitation (rainfall), and relative humidity at 9:00 (RH 0900) and 15:00 (RH 1500). CliMond (http://www.climond.org/, accessed on 22 May 2024) is a global climatology resource designed for bioclimatic modeling, offering gridded climate data layers of history and climate scenarios in the future at 10’ or 30’ spatial resolutions [33]. The standard format required for CLIMEX modeling is available for download from this platform. In this study, we selected 10’ climate scenario layers to enhance result accuracy. Historical meteorological data from 1961 to 1990 (with a 30-year focus around 1975) were obtained from the WorldClim and Climate Research Unit (CRU) datasets (CL1.0 and CL2.0). Future meteorological data were sourced from the Global Climate Model (GCM) data presented in the IPCC Fourth Assessment Report (AR4) [34]. Four emission scenarios—A1, A2, B1, and B2—are included in IPCC AR4. The A1 storyline and scenario family describe a future world of rapid economic growth, a global population that peaks in the mid-century and declines thereafter, and the rapid introduction of new and more efficient technologies. The A1 scenario family develops into three groups that describe alternative directions of technological change in the energy system. The three A1 groups are distinguished by their technological emphasis: fossil energy-intensive (A1FI), nonfossil energy sources (A1T), and balance across all sources (A1B). Here, the medium and highest greenhouse gas emission scenarios, A1B and A2, were selected to predict suitable climatic conditions for *A. eugenii*. To achieve clearer results, the historical and 2090 climate data were selected to predict the potential distribution of *A. eugenii*.

For RF modeling, the necessary bioclimatic layers can be freely accessed from WorldClim (https://www.worldclim.org/, accessed on 22 May 2024). A total of 19 bioclimatic layers, available in resolutions of 0.5’, 2.5’, 5’, or 10’, can be downloaded. To align with the resolution used in CLIMEX modeling, we selected 10’ bioclimatic layers for this study. Historical climate data from 1961 to 1990 (a 30-year period centered on 1975) were interpolated using the thin plate smoothing spline function applied to observed data from meteorological stations worldwide [35]. In the sixth IPCC report, Shared Socioeconomic Pathways (SSPs) were proposed based on different trends in socioeconomics, population, technology, lifestyles, policies, institutions, and other factors [36]. The SSPs are divided into five pathways: SSP1, SSP2, SSP3, SSP4, and SSP5. Future climate data for SSPs are typically generated using global climate models that have been scaled down and calibrated (deviation-corrected) [35]. The SSP3-7.0 and SSP5-8.5 scenarios represent the medium- and worst-case outcomes of future climate change, respectively. Here, to be essentially consistent with climate scenarios in CLIMEX modeling, the historical climate data and SSP3-7.0 and SSP5-8.5 climate data in 2090 were selected to project the distributions of *A. eugenii* and its hosts in RF, respectively.

### 2.4. Using RF to Project the Potential Global Distribution of A. eugenii, C. annuum, and C. frutescens

#### 2.4.1. Modeling Process

In this study, the RF model from the BioMod2 package in R (R Foundation for Statistical Computing, Vienna, Austria) was used to predict the potential distributions of *A. eugenii*, *C. annuum*, and *C. frutescens* [37,38]. To reduce sampling bias caused by occurrence records from different sources, pseudo-presence points were randomly generated, 4-fold the number of presence points [39]. The model was trained and validated using 10-fold cross-validation. In each fold, one subset was retained as validation data to test the model, while the remaining subsets were used for training. For each subset, 70% of the occurrence data was used to train the model, and the remaining data was used to assess its predictive performance. The model was run 10 times for cross-validation, enhancing accuracy, minimizing errors, and providing more realistic predictions. The significance of environmental variables influencing the distribution of *A. eugenii* was assessed through corresponding statistical analyses. This methodology produced more accurate and realistic distribution maps for *A. eugenii*, *C. annuum*, and *C. frutescens*.

#### 2.4.2. Evaluation of Model Accuracy

Model accuracy under each climate scenario was assessed using the area under the curve (AUC), true skill statistic (TSS), and kappa values. The AUC represents the area under the receiver operating characteristic (ROC) curve, with values ranging from 0 to 1 [40]. The kappa coefficient evaluates the classification accuracy of the model based on the confusion matrix, with a range from −1 to 1 [41]. The TSS, an enhanced index derived from the kappa coefficient, measures the model’s ability to discriminate between positive and negative classes by calculating the difference between true and false positive rates, with values ranging from 0 to 1 [42]. Higher values of these three indices (closer to 1) indicate better model accuracy.

#### 2.4.3. Classification of Suitability

The suitability levels in a study area are typically classified to distinguish the suitability of a species in different regions more intuitively. The RF model prediction results comprised probability grids ranging from 0 to 1. Different suitability levels can be obtained by setting thresholds according to the observed distribution of the species. For *A. eugenii*, we first used the natural breaks (Jenks) method in ArcGIS 10.8 (Environmental System Research Institute Inc., Redlands, CA, USA) to preliminarily divide suitability levels into four categories: unsuitable, low suitability, medium suitability, and high suitability. The natural breaks (Jenks) method adheres to the principle of maximizing intergroup variance while minimizing intragroup variance, which results in good classification performance [43]. Next, because unsuitable areas represented areas where the species could not survive, the unsuitable range was adjusted to 0. Finally, based on the principle that areas with denser *A. eugenii* records have higher suitability, the thresholds for other suitability levels were adjusted, resulting in the final classification. For *C. annuum* and *C. frutescens*, their suitability was classified into suitable (probability greater than 0) and unsuitable (probability equal to 0) categories, which were processed in subsequent steps.

### 2.5. Using CLIMEX to Project the Potential Global Distribution of A. eugenii

#### 2.5.1. CLIMEX Model

CLIMEX was employed to predict the global distribution of *A. eugenii* by creating semi-mechanistic models that simulate the species’ response to climatic conditions [26,44]. In CLIMEX, the Ecoclimatic Index (EI) is used to indicate the distribution potential of a species at a site. The EI value was determined using the annual growth index (${\mathrm{GI}}_{A}$), stress index (SI), interaction stress index (SX), and two limiting conditions: sufficient degree days to complete the life cycle (PDD) and the obligate diapause index (DI). The EI ranges from 0 to 1, with higher values indicating a higher likelihood of a species being present, and it can only reach 100 under ideal conditions. The formula used to calculate EI is as follows (SX is usually not considered):

$$\mathrm{EI}\;=\;{\mathrm{GI}}_{A}\;\times \;\mathrm{SI}\;\times \;\mathrm{SX}$$

${\;\mathrm{GI}}_{A}$ reflects the potential for population growth under suitable growth conditions and is primarily affected by the temperature index (TI) and the moisture index (MI). The formula is as follows:

$${\mathrm{GI}}_{A}\;=\;\mathrm{TI}\;\times \;\mathrm{MI}$$

Stress indices, including cold stress (CS), heat stress (HS), dry stress (DS), and wet stress (WS), demonstrate the degree of population decline during unfavorable seasons. The formula used is as follows:

$$\mathrm{SI}\;=\;\left(1\;-\frac{\mathrm{CS}}{100}\right)\;\times \;\left(1\;-\;\frac{\mathrm{HS}}{100}\right)\;\times \;\left(1\;-\;\frac{\mathrm{DS}}{100}\right)\;\times \;\left(1\;-\;\frac{\mathrm{WS}}{100}\right)$$

#### 2.5.2. Parameter Fitting

The specific parameters were set according to the following methods. First, suitable templates were selected, and the initial parameter values were set based on the climate type of the native region (North and Central America and the Caribbean) and the biological data of *A. eugenii*. Next, 75% of the global occurrence records were randomly selected as the training set, serving as the foundation for parameter adjustment [45]. These parameters were iteratively refined to align the model’s projected potential distribution with the known distribution records as closely as possible. After parameter optimization, the remaining occurrence records were used to evaluate the model. The detailed basis for fitting each parameter is outlined below, and the final parameter values are presented in Table 1.

**Temperature index (TI):** TI consists of four temperature parameters: lower temperature threshold (DV0), lower optimum temperature (DV1), upper optimum temperature (DV2), and upper temperature threshold (DV3), which can be set with reference to experimental measurements and the actual geographical distribution of the species. Toapanta et al. found that the lower temperature threshold for *A. eugenii* to develop from egg to adult was 9.6 °C; therefore, DV0 was set at 9.6 °C [46]. In terms of optimal temperature for *A. eugenii*, Toapanta et al. considered it to be 30 °C, while it was 29 ± 3 °C or 31 ± 1 °C in the study of Rossini et al. [47]. Therefore, DV1 and DV2 were set at 28 °C and 31 °C, respectively. DV3 was set at 33 °C based on a decline in the developmental rate of *A. eugenii* at 33 °C, as reported by Toapanta et al. [46].

**Moisture index (MI):** MI comprises the lower soil moisture threshold (SM0), lower optimal soil moisture (SM1), upper optimal soil moisture (SM2), and upper soil moisture threshold (SM3). Zhu et al. showed that the optimal field moisture capacity for the growth of *C. annuum*, one of the most important hosts of *A. eugenii*, is 70–85%, and SM1 and SM2 for *A. eugenii* were set to 0.7 and 0.85, respectively [48]. Based on the temperate and semi-arid template data in CLIMEX and the geographical distribution of *A. eugenii*, SM0 and SM3 were set at 0.1 and 1.5, respectively, to achieve the best model fit.

**Cold stress (CS):** CS indicates the minimum daily thermal accumulation required for species survival, defined by the cold stress temperature threshold (TTCS) and cold stress temperature rate (THCS). Fernández et al. found that adult *A. eugenii* are unlikely to overwinter in regions of Canada that consistently experience temperatures below −10 °C [9]. Consequently, a TTCS of −10 °C and THCS of −0.01 week^−1^ were set so that areas with EI > 0 cover the two occurrence records in Canada and the Netherlands, respectively.

**Heat stress (HS):** HS is determined by the heat stress temperature threshold (TTHS) and heat stress temperature rate (THHS). HS can only begin to accumulate at temperatures greater than or equal to DV3, so TTHS should be greater than or equal to 33 °C. In the semi-arid template, TTHS and THHS were 39 °C and 0.002 week^−1^, respectively, and to match the distribution records of *A. eugenii* in the Mexican semi-arid region, TTHS and THHS were set at 41.7 °C and 0.005 week^−1^, respectively.

**Dry stress (DS):** DS is determined by the dry stress threshold (SMDS) and dry stress rate (HDS). SMDS is to be less than or equal to SM0, and by continuously iterating the model, finally SMDS and HDS are set to 0.1 and −0.005 week^−1^, respectively, to match the predicted results with the *A. eugenii* realistic distribution.

**Wet stress (WS):** WS is determined by the wet stress threshold (SMWS) and wet stress rate (HWS). In the temperate and semi-arid templates, the SMWS is 2.5 and 0.4, respectively, and the HWS is 0.002 week^−1^ and 0.01 week^−1^, respectively. Because the global distribution range of *A. eugenii* is between these two climates, its SMWS and HWS were set at 2 and 0.001 week^−1^, respectively, to align the model results as closely as possible to the known distribution.

**Degree days per generation (PDD):** PDD represents the effective accumulated temperature (degree days) for a species to complete the development of one generation, with temperatures exceeding DV0. According to Toapanta et al., the development of *A. eugenii* from egg to adult requires 256.4 °C days; therefore, PDD was set at 256.4 °C days [46].

**Diapause index (DI):** Diapause is an adaptive strategy used to tolerate unsuitable environmental conditions. No diapause has been detected in *A. eugenii* to date; thus, the DI was not set.

#### 2.5.3. Classification of EI Values

To distinguish the suitability of a species in different regions more intuitively, the range of EI values of the species is typically classified into different suitability levels [49]. First, based on the definition of EI, areas where EI = 0 were classified as unsuitable for the species. The low-, medium-, and high-suitability areas were delineated based on the density of *A. eugenii* distribution records and the corresponding EI values in the United States and Central America. Because *A. eugenii* is densely distributed across Central America and the Florida Peninsula, areas with tropical rainforests, tropical savannas, subtropical monsoons, or subtropical humid climates are classified as high-suitability zones. Therefore, the range for high-suitability areas was set to EI > 12, thus ensuring that these regions were classified as highly suitable. In northern and western North America, the distribution records become sparser, as most of these regions have temperate continental climates. Based on this pattern, the EI value ranges for medium- and low-suitability areas were set at 5 < EI ≤ 12 and 0 < EI ≤ 5, respectively, to match the actual distribution of *A. eugenii*.

### 2.6. Potential Global Distribution of A. eugenii Hosts

Using ArcGIS, the potential distributions of *C. annuum* and *C. frutescens* were reclassified, with grids having values greater than 0 assigned a value of 1 and grids with a value of 0 assigned a value of 0. This resulted in binary raster maps of *C. annuum* and *C. frutescens*. Binary rasters of *C. annuum* and *C. frutescens* were overlaid to create a raster map with values of 0, 1, or 2. Areas with a value of 1 or 2 were classified as suitable, whereas those with a value of 0 were classified as unsuitable. This process yielded a global distribution map of *A. eugenii* hosts.

### 2.7. Potential Global Distribution of A. eugenii Considering Its Hosts

In ArcGIS, suitable areas from the potential global host distribution of *A. eugenii* were used as masks to extract its potential global distribution as predicted by RF and CLIMEX, resulting in a host-considered potential global distribution.

### 2.8. Potential Global Distribution of A. eugenii Predicted Using the Ensemble Model

The next step was performed in ArcGIS using the layers described in Section 2.7. Each suitability level layer from the CLIMEX results was used as a mask to extract the RF result layers. Since each CLIMEX suitability level layer can extract four types of suitability level areas in RF, this process resulted in 16 types of areas with different suitability levels, represented by the ensemble model, which combines RF and CLIMEX.

## 3. Results

### 3.1. Global Hosts’ Potential Distribution for A. eugenii Projected Using RF

**Figure 2:**
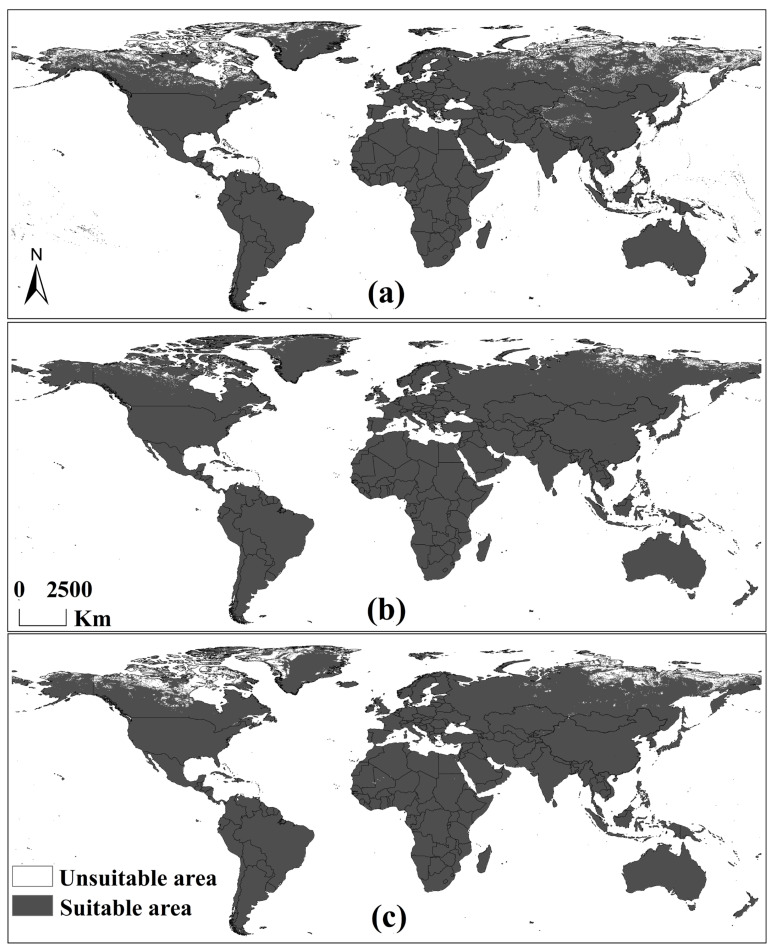
Potential global distribution of *C. annuum* and *C. frutescens*: (**a**) in the historical climate scenario in 1975; (**b**) in the SSP-370 scenario in 2090; (**c**) in the SSP-585 scenario in 2090.

The evaluation indicators for the RF modeling accuracy of *C. frutescens* and *C. annuum*—AUC, kappa, and TSS—in each climate scenario are shown in Table 2, indicating sufficient modeling accuracy. Under historical climate scenarios, the hosts were distributed globally, except in some northern regions of Canada and Russia, as well as in some areas of the Tibetan Plateau in China (Figure 2). Under the SSP3-7.0 and SSP5-8.5 climate scenarios, the hosts were distributed globally, except in the northern regions of Canada and Russia. Overall, the distribution area of *A. eugenii* hosts showed a northward expansion trend in the Northern Hemisphere, which was more pronounced under the SSP3-7.0 climate scenario.

### 3.2. Potential Global Distribution of A. eugenii Projected Using RF Considering the Hosts

#### 3.2.1. Model Performance and Key Environmental Factors

**Figure 3:**
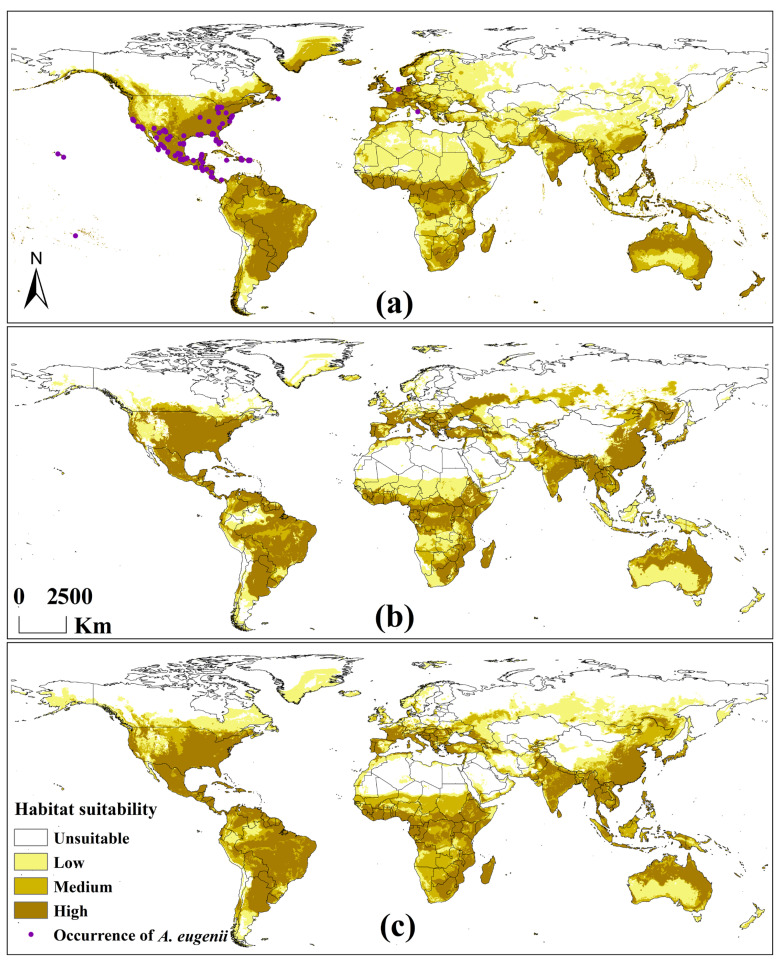
Potential global distribution of *A. eugenii* predicted by RF: (**a**) in the historical climate scenario in 1975; (**b**) in the SSP-370 scenario in 2090; (**c**) in the SSP-585 scenario in 2090.

**Figure 4:**
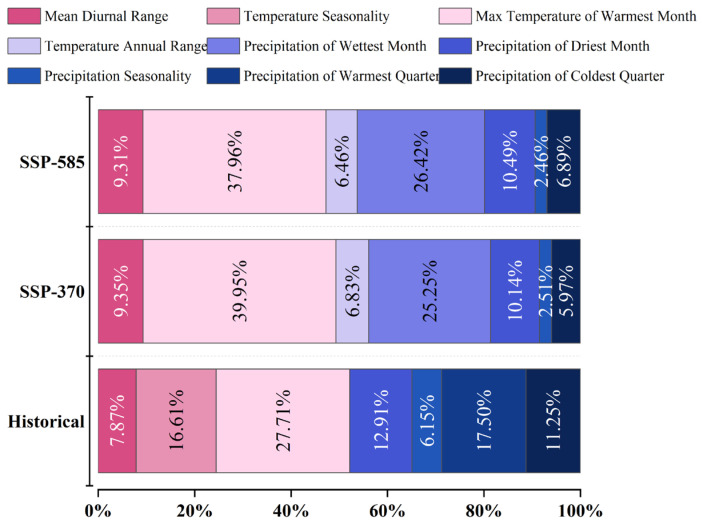
Variables’ contribution in the prediction about *A. eugenii* by RF.

The values of the model evaluation indicators—AUC, kappa, and TSS—for *A. eugenii* in each climate scenario indicated that our model had sufficient accuracy (Table 2). All records were distributed within a suitable area, further demonstrating the accuracy of the model (Figure 3a). The contributions of the key environmental factors to the model results for each climate scenario are shown in Figure 4. Under the three climate scenarios, four temperature and five precipitation variables were selected as key environmental factors. The contribution of temperature variables was slightly greater than that of the precipitation variables, with the maximum temperature in the warmest month being the most significant variable. This suggests that *A. eugenii* is more sensitive to temperature than precipitation, providing valuable insights into how *A. eugenii* may respond to climate change.

#### 3.2.2. Potential Global Distribution Under Historical Climate Scenario

Under the historical climate scenario, *A. eugenii* is found globally, except in regions at higher latitudes or altitudes in the Northern Hemisphere (Figure 3a). The areas with high suitability for *A. eugenii* are roughly as follows: North America: The entire Central America region; the southern, northeastern, and western coastal regions of the United States; Atlantic Canada; and the southern tip of Greenland. South America: Orinoco Plain, Guiana Highlands, Brazilian Highlands, La Plata Plain, Paraná Highlands, and the surrounding areas. Europe: Western Europe, western Central Europe, western Southeastern Europe, northern Southern Europe, and western coastal and surrounding regions of Norway. Africa: Low-latitude regions of West, Central, and East Africa; southwestern coastal Africa and surrounding regions; Madagascar. Asia: Southern China, Japan, India, surrounding regions, and most of Southeast Asia. Oceania: The coastal and northern regions of Western Australia, northern Australia, Queensland, New South Wales, Victoria, Tasmania, and New Zealand.

#### 3.2.3. Potential Global Distribution Under Future Climate Scenarios

Under the 2090 SSP3-7.0 and SSP5-8.5 climate scenarios, the potential distribution areas of *A. eugenii* showed the same trend of change as in the historical climate scenario (Figure 3b,c). North America: *A. eugenii* will continue to spread northward, whereas areas originally suitable in Canada and Greenland will become unsuitable. South America: The suitable area will decrease slightly and will be mainly concentrated in the Amazon Basin. Europe: Originally suitable areas in Western and Northern Europe will become unsuitable, with a noticeable shift in suitable areas toward the east. Africa: Subtropical and tropical desert climatic areas will become completely unsuitable for *A. eugenii*, whereas areas to the south of these regions will remain suitable. Asia: Many regions in Western and Central Asia will become unsuitable, whereas the suitability of *A. eugenii* in Northern Asia will increase to some extent. China: The suitability for *A. eugenii* will significantly increase, spreading toward the north and northeast, but will decrease in the northwest. Oceania: The suitability for *A. eugenii* in Australia will slightly decrease, whereas that in New Zealand will significantly decrease. The main difference between the SSP3-7.0 and SSP5-8.5 climate scenarios was mainly reflected in the fact that SSP3-7.0 predicts more areas with newly added high suitability, while SSP-585 predicts more medium- and low-suitability areas. Overall, the suitable area under SSP5-8.5 was larger than that under SSP3-7.0.

### 3.3. Potential Global Distribution of A. eugenii Projected Using CLIMEX Considering the Hosts

#### 3.3.1. Model Performance

**Figure 5:**
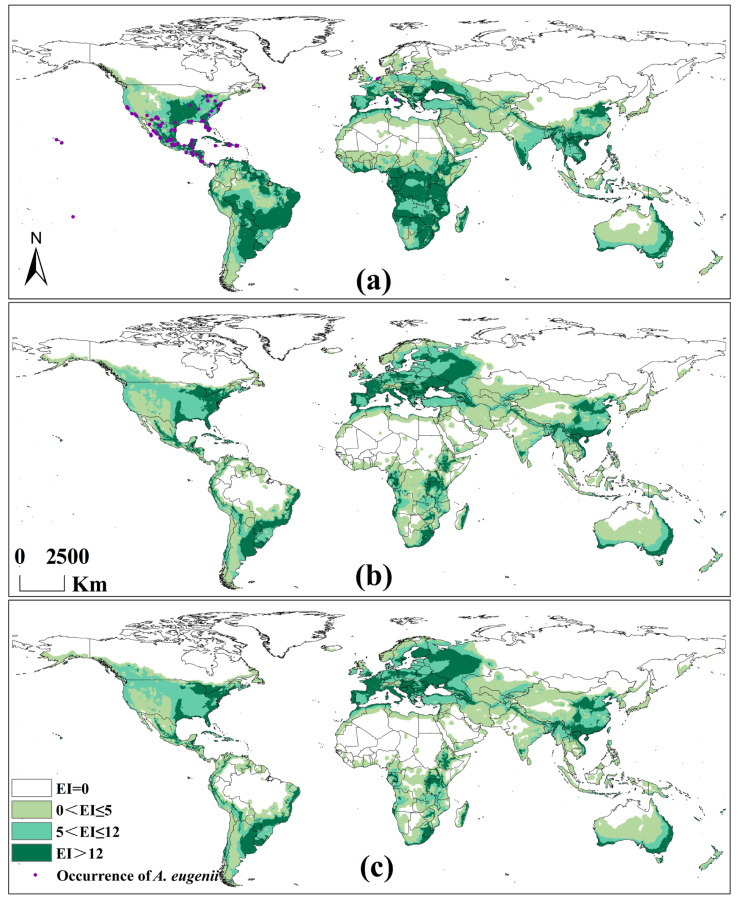
Potential global distribution of *A. eugenii* predicted by CLIMEX: (**a**) in the historical climate scenario in 1975; (**b**) in the A1B scenario in 2090; (**c**) in the A2 scenario in 2090.

An important method for assessing the accuracy of the final parameters in CLIMEX is to examine the spatial relationship between the predicted areas and globally recorded records of *A. eugenii*. Our results showed that 100% of the recorded records were within the potential distribution predicted by CLIMEX. Areas with denser records had high EI values, whereas those with sparser records had low EI values (Figure 5a). These results indicated that the final parameters of the model were reliable.

#### 3.3.2. Potential Global Distribution Under Historical Climate Scenario

Under the historical climate scenario, *A. eugenii* adapted to various climate types and is widely distributed globally, except in regions with tropical deserts and temperate continental and polar climates (Figure 5a). The highly suitable areas for *A. eugenii* are roughly distributed as follows: North America, most areas of the Mississippi Plain and Florida Peninsula in the United States, and most areas of Central America. South America: Mainly distributed in the Guiana Highlands, Brazilian Highlands, and the La Plata Plain. Europe: The region north of the Mediterranean and Black Sea coasts. Africa: A small part of the Southern Mediterranean coast, the Azande Plateau, the East African Rift Valley, and some areas of Southern Africa and Madagascar. Asia: Northern regions of China, areas around and South of the Tropic of Cancer, a small part of the region South of the Himalayas, the Deccan Plateau in India, Sri Lanka, the Indochina Peninsula, the Philippines, and Southern Malaysia. Oceania: Queensland, New South Wales, Victoria, and coastal and nearby areas of Western and Southern Australia.

#### 3.3.3. Potential Global Distribution Under Future Climate Scenarios

Under the 2090 A1B and A2 climate scenarios, the potential distribution areas of *A. eugenii* show the same trend of change as in the historical climate scenario (Figure 5b,c). North America: Suitable areas show an expanding trend toward the northeast and northwest. Several states in the Midwest, Northeast, and South will become new high-suitability areas for *A. eugenii*. However, its suitability in Central America will decrease. South America: The suitability of *A. eugenii* will decrease significantly, mainly reflected in the noticeable reduction in high-suitability areas. Some areas originally suitable in the Orinoco Plain, Amazon Basin, and Brazilian Highlands will become completely unsuitable. Europe: An overall trend of the northward expansion of suitable areas will occur, with high-suitability areas extending outward from their original locations. Suitability in the western regions of Russia is expected to increase significantly. Africa: The total suitable area south of the Saharan Desert will decrease significantly, with the originally continuous suitable areas becoming fragmented. Only a few areas will maintain high suitability. Asia: An overall trend of northward migration of suitable areas will occur, reflected in reduced suitability in Western Asia, Southeast Asia, and South Asia, whereas suitability in Central and East Asia will increase. In China, the suitability of *A. eugenii* will increase in the southwestern and northwestern regions. Oceania: No significant changes are expected in the distribution of suitable areas for *A. eugenii* in Oceania. The main difference between the A1B and A2 climate scenarios is that in the A2 scenario, suitable areas for *A. eugenii* extend further north in the Northern Hemisphere, and suitable areas in Africa become increasingly fragmented.

### 3.4. Potential Global Distribution of A. eugenii Projected Using Ensemble Model

#### 3.4.1. Potential Global Distribution Under Historical Climate Scenario

**Figure 6:**
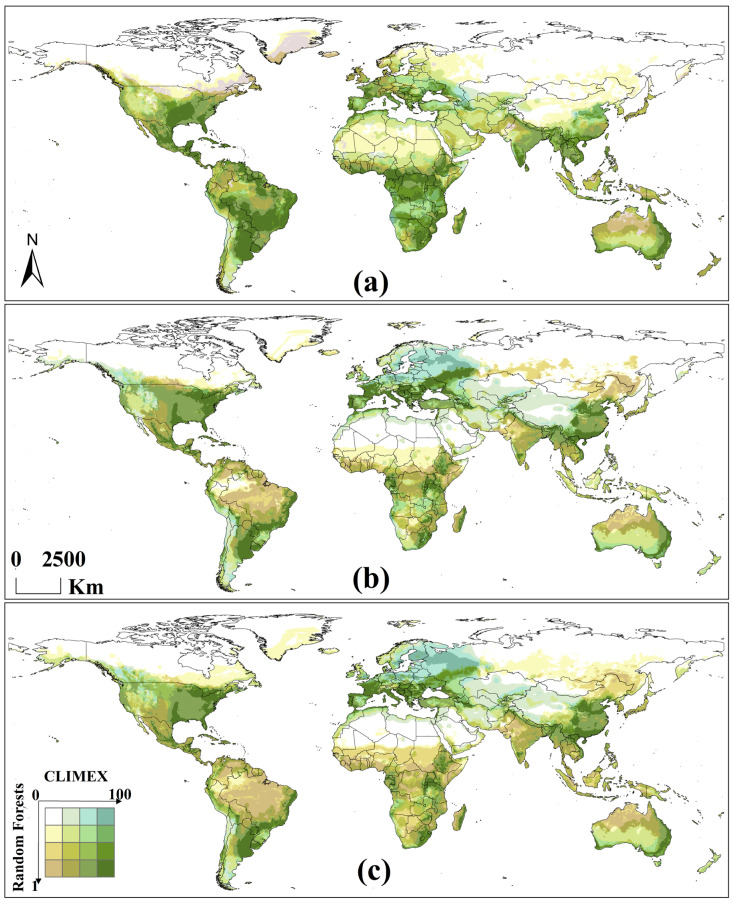
Potential global distribution of *A.eugenii* predicted by the ensemble model: (**a**) in the historical climate scenario in 1975; (**b**) in the SSP-370 and A1B scenarios in 2090; (**c**) in the SSP-585 and A2 scenarios in 2090.

The suitability of *A. eugenii* was predicted using the ensemble model for each climate scenario (Figure 6). The color of each grid cell on the map represents both the suitability level predicted using CLIMEX and that predicted using the RF model. Under the historical climate scenario, the predictions of the ensemble model were as follows: In North America, the results from the two models were quite similar. Both models indicated that the Mississippi Plain, Florida Peninsula, and most of Central America are suitable for *A. eugenii* survival (Figure 6a). In contrast, the RF model predicted that more northern regions would still be suitable. South America: Both models agree that most of South America is suitable for *A. eugenii*, with many areas in the Brazilian Highlands and La Plata Plain being highly suitable for both models. Europe: The high-suitability areas identified by both models were mostly along the Mediterranean and Black Sea coasts. In contrast, the RF model indicated that northern regions are more suitable. Africa: Both models agreed that high-suitability areas are concentrated along the southern Mediterranean coast, Central and Southern Africa, and Madagascar. In contrast, the RF model suggested that *A. eugenii* has low suitability in the Saharan Desert. Asia: Both models agree that high-suitability areas are concentrated in southern India, Sri Lanka, a small portion of the region south of the Himalayas, the low-latitude regions of China, the Indochina Peninsula, and the Philippines. In contrast, the RF model suggested that more northern regions still show suitability and that the suitability range in Malaysia is larger. Oceania: Both models indicated that suitable areas are concentrated along the eastern coast of Australia and extend inland. In contrast, the RF model suggested that some northern regions are still suitable.

#### 3.4.2. Potential Global Distribution Under Future Climate Scenarios

Under the two future climate scenarios, the trends in suitable areas were similar (Figure 6b,c). North America: In the United States, both models predicted that high-suitability areas would shift northeastward, with the overall suitable area expanding northward. Therefore, the suitability of *A. eugenii* in Central America will decrease. South America: Both models predicted that the high-suitability area would shrink, with suitable areas remaining only on the La Plata Plain and some eastern mountain ranges. The RF model indicated that the Brazilian Highlands will still be highly suitable for *A. eugenii*. Europe: Both models predicted that high-suitability areas would expand outward from their original locations, with a noticeable trend toward expansion into western Russia. CLIMEX predicted that areas suitable for *A. eugenii* will expand further north. Africa: Both models predicted a significant reduction in high-suitability areas. The RF model suggested that sub-Saharan Africa will retain relatively high suitability. Asia: Both models predicted an increase in high-suitability areas in China with outward expansion from the original areas. However, suitability will decrease in the Indochina Peninsula and India. In most parts of Saudi Arabia, *A. eugenii* will no longer be suitable. Suitable areas will generally shift northward, with the RF model predicting a more northerly trend. Oceania: The joint predictions for Oceania from both models showed no significant changes compared with those of the historical scenario, with the main difference being that the RF model predicted that Tasmania and New Zealand would no longer be suitable for *A. eugenii*. The main difference between the two climate scenarios is that the high-emission scenarios (SSP3-7.0 and A2) predict a larger area of suitable zones and a stronger overall suitability than the low-emission scenarios (SSP5-8.5 and A1B).

#### 3.4.3. Distribution Area of Different Suitability Levels

**Figure 7:**
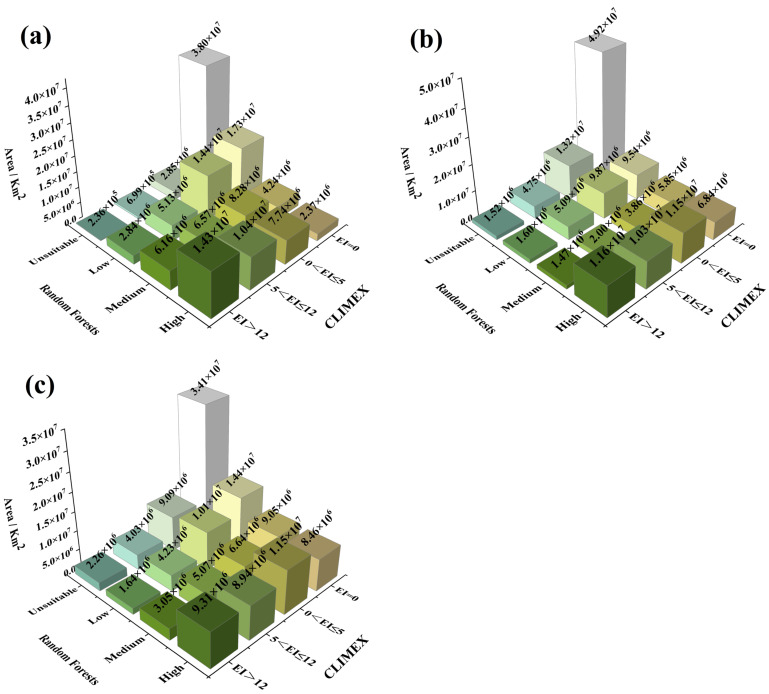
Potential global distribution areas of *A.eugenii* based on the ensemble model: (**a**) in the historical climate scenario in 1975; (**b**) in the SSP-370 and A1B scenarios in 2090; (**c**) in the SSP-585 and A2 scenarios in 2090.

The specific areas of the 16 suitability levels predicted using the ensemble model for each climate scenario. In all climate scenarios, both models agreed that the area of unsuitable regions was the largest among all suitability levels (Figure 7). The total areas of the 15 levels suitable for *A. eugenii* across history and low-emission and high-emission climate scenarios represented 73.12, 66.82, and 75.97%, respectively, of the total land area of the six continents (excluding Antarctica). Both models predicted that the area of high suitability decreased by 19.05 and 35.02% under the low- and high-emission climate scenarios, respectively, compared with that in the historical scenario. The proportion of the total suitable area decreased slightly under the low-emission scenario compared with that under the historical scenario, but the reduction in high-suitability areas was smaller than that under the high-emission scenario. Under the high-emission scenario, the total suitable area increased compared with that under the historical scenario, but the area of high suitability decreased more significantly than that under the low-emission scenario.

## 4. Discussion

Climate change and invasive alien species are major issues currently facing humanity and are related to the economic development and ecological security of all countries [50]. *A. eugenii* can only spread naturally over limited distances; however, it can be transported internationally via Capsicum fruits and possibly aubergines. Frequent international trade and transportation are required to accelerate the spread of this pest [51]. Our prediction map clearly indicates the potential distribution and transmission routes of *A. eugenii* worldwide, providing a basis for quarantining and controlling this pest.

### 4.1. Potential Global Distribution Range Changes of A. eugenii Under Historical and Future Climate Conditions

The climatically suitable areas for *A. eugenii* are mainly distributed in the Midwestern and Southern United States, Central America, the La Plata Plain, parts of the Brazilian Plateau, the Mediterranean and Black Sea coasts, sub-Saharan Africa, Northern and Southern China, Southern India, Indochina Peninsula, and eastern coastal Australia. Several regions exhibit apparent alterations under the future climate scenarios. In the Northern Hemisphere, suitable areas in North America, Europe, and China are projected to expand toward higher latitudes. In China, the number of highly suitable areas is expected to significantly increase, mainly in the south and north. Conversely, suitable areas in Central America, northern South America, India, and the Indochina Peninsula will become less suitable. These findings provide important insights into the global spread of this pest via human transportation of infected hosts. Taking the chili industry in China as an example, we calculated the potential economic losses caused by *A. eugenii*. These losses were determined by multiplying the production value of chili by the suitability index and the loss coefficient; the production value of chili was obtained from the average production value of chili in China from 1991 to 2020, which was 799 million yuan (FAO, https://www.fao.org/faostat/en/#data/QV, accessed on 2 June 2024). The suitability index was obtained from the average suitability index calculated using the ensemble model. Briefly, a grid transformation was performed on the distribution layer of suitable habitats of *A. eugenii* in China. The grid-to-point function in SDM Tools (http://www.sdmtoolbox.org, accessed on 2 June 2024) was used to obtain a grid map of the point features, then view the attribute table of the point features and check the suitability index [52]. Finally, we took the average of all the suitability indices in China. The average suitability index was 0.165. The loss coefficient was derived from the average yield loss rate of chili of 0.612 reported worldwide. According to calculations, the potential economic loss caused by *A. eugenii* to the chili industry in China each year can reach 81 million yuan. Hence, strict quarantine is a top priority for preventing the continued global spread of *A. eugenii*.

### 4.2. Major Climatic Factors Affecting the Potential Global Distribution of A. eugenii

Our results indicate that temperature plays a dominant role in the potential distribution of *A. eugenii* on the global scale. Under the three climate scenarios, the cumulative contribution of the temperature variables exceeded that of the precipitation variables. Among the top three temperature variables in terms of contribution, the maximum temperature in the warmest month was selected as the key variable affecting the potential distribution of *A. eugenii*, with an average contribution rate of over 35%. Insects are poikilotherms influenced by changes in temperature, which is the most important environmental factor affecting their biology, including their growth and development rates, number of populations produced, and geographical distribution [19]. *A. eugenii* usually takes 20–30 days to complete one generation and develops faster in summer, reaching up to eight generations per year [9]. Under normal conditions, 3–5 generations are completed annually. In addition, this pest also has no diapause. Some studies have shown that high temperatures in summer are the direct reason for accelerating the growth and development of *A. eugenii*, resulting in more generations [46,47]. Changes in suitability resulting from climate change vary among regions. Overall, the distribution of this pest in the Northern Hemisphere is expected to expand to higher-latitude regions. Moreover, the colonial history of this pest from Central and Northern America to the high-latitude regions of North America also proves that with the continuous rise in global temperatures, the northern boundary limiting its geographical distribution has moved toward high-latitude regions. An evident temperature change phenomenon in the Northern Hemisphere is the rise in summer temperatures [53]. Therefore, the current increase in global temperature has broadened the living conditions and geographical distribution of this pest, greatly increasing the probability of population survival. These findings may help identify regions highly sensitive to local climate changes and characterize suitability changes under climate change scenarios. The results of this study provide guidance for the prevention and control of *A. eugenii*.

### 4.3. Quarantine Measures and Management Plan for A. eugenii

Quarantine supervision needs to be strengthened globally [54]. Phytosanitary conditions are an effective measure to prevent the spread of *A. eugenii* [55]. Enhanced quarantine inspection and occurrence monitoring should be implemented for pepper-related products imported from countries where the pest is known to occur. Once *A. eugenii* is intercepted in goods, corresponding quarantine measures should be taken immediately for imported goods, such as destruction or return [56].

The management and regular risk assessments of *A. eugenii* should be augmented [54]. Based on the global population dynamics of *A. eugenii*, timely quantitative risk assessments, such as suitability, adaptability, and potential spread direction, should be conducted, and corresponding management measures should be adjusted promptly. Particularly in highly suitable regions, strengthening pest monitoring can facilitate early detection and eradication.

The forecasting of *A. eugenii* populations globally needs strengthening [9]. Governments and international organizations should establish a customized monitoring system for *A. eugenii* globally, focusing on forecasting international development trends of the pest. This will enable the formulation of appropriate prevention plans and control measures.

Publicity efforts should be intensified, and global prevention awareness should be increased. Widely publicizing the hazards of indiscriminately transporting pepper-related products via media and promoting the importance of plant quarantine work will lay a solid foundation for smoothly implementing plant quarantine and pest control efforts.

Continuously increasing investments in phytosanitation, improving relevant policy measures, reinforcing quarantine team development, and providing relevant technical training for inspection and quarantine department staff are crucial. Relevant government departments should coordinate and cooperate to prevent the invasion of *A. eugenii*, such as by organizing international exchanges and cooperation for the census of this pest. Vigorously promoting ecological and biological control technologies for this pest by integrating physical, chemical, and biological control measures to comprehensively prevent and control *A. eugenii* is also necessary [7,57].

### 4.4. Limitations and Future Prospects

Our study has some limitations. First, niche conservatism must be seriously considered. Niche conservatism refers to the fact that the realized niche of a species does not undergo significant changes over time or spatial transformations [58]. This is an important prerequisite for using SDMs to predict the probability of species distribution at different times or geographical spaces. Therefore, SDMs often assume that the relationship between species and environmental conditions is stable; however, in reality, species have strong adaptability to new environments [59]. If the species fitted in the model has a non-stable relationship with the environment, once environmental conditions change, organisms may adjust to suitable habitats, leading to deviations in the predictions of the original model [60]. The emergence of non-stable relationships between species and the environment is largely because almost all modeling data for SDMs come from realized niches rather than theoretical ones (including fundamental and realized niches). Furthermore, various factors that shape species distribution patterns, such as biological interactions, behavior, and human activity, are difficult to fully incorporate into model construction. In addition, the limiting factors of model performance may be complex and include the non-conservativeness of biological and environmental responses, generalization of resource utilization, incomplete niche description, changes in ecosystem state, lack of important influencing factors, biological interactions, observation bias, population fluctuations, model overfitting, violation of assumptions, and non-equilibrium distribution [61]. Indeed, driving processes include environmental fluctuations, external disturbances, dispersion, migration, and diffusion. Moreover, the niche conservatism hypothesis ignores intraspecific variations in species. Recently, numerous studies have shown that the ecological niche of invasive species shifts in the regions they have invaded. The rapid environmental adaptation of alien species plays a crucial role in the invasion process, leading to significant differentiation between different geographic populations [62]. Fully considering these factors in SDMs is a long-term and arduous challenge. However, based on the existing research foundation, gradually integrating physiological mechanisms, life history stages, population dynamics, and unstable relationships into existing SDMs may be the focus of future research.

Second, the transferability of the model must be considered carefully. This refers to the predictive ability of a model in a new space or time outside the environment in which the data were modeled [63]. Although the evaluation of model transferability relies on spatially and temporally independent species distribution data because of the small amount of available data, current research often uses a cross-validation method to assess the credibility of model prediction results [64]. Data within the same spatiotemporal range are divided into two parts according to a certain proportion: one part is used for modeling, and the other is used for model evaluation. The modeling and evaluation data of cross-validation are not truly independent in time and space, which limits the analysis and understanding of model transferability [65]. However, biological invasion provides ideal data for evaluating model transferability because the distribution data of alien species in their place of origin and invasion are independent [66]. To explore the overall impact of ecological niche changes on the transferability of SDMs, Liu et al. integrated data from 235 invasive species from existing studies and found that model transferability was closely related to the degree of ecological niche changes in species in their place of origin and invasion [63]. When invasive species occupy similar environments in two locations (i.e., with larger stable parts), the transferability of the model is significantly higher. When a species has a wide range of suitable environments that are not occupied in the invaded area (i.e., a large missing part), the transferability of the model is low. In addition, many studies have pointed out that model transferability is closely related to factors such as the algorithms used, ecosystem characteristics, environmental factors, and spatiotemporal scope [67]. Considering the impact of the types of environmental variables, number of predictive factors, and shape of species response curves (such as linear) on model transferability, users should choose algorithms with appropriate complexity and carefully consider the selection and setting of environmental factors and model structures during the construction and application of the model.

Finally, alternative hosts and anthropogenic factors need special attention, as they may impose constraints on niches and realized distributions. First, *A. eugenii* has many host plants, far beyond *C. annuum* and *C. frutescens* [9]. In addition to *Capsicum*, plants of the genera *Physalis*, *Lycopersicon*, *Datura*, *Petunia*, and *Nicotiana* are also fed on by *A. eugenii*. Moreover, many plants of the genus *Solanum* can also be fed on by this pest [1]. For instance, *A. eugenii* has been found on *Solanum hindsianum* plants around pepper plantations in Baja California Sur and Mexico. In addition, *Solanum elaeagnifolium* is now recognized as the main alternative host of *A. eugenii*. *A. eugenii* can also feed on *Petunia philadelphica* [47]. During the non-planting period, naturally growing *Solanum* plants around pepper fields provide food or spawning grounds for *A. eugenii*, allowing its population to persist [6]. These *Solanum* plants generally grow around cultivated fields before pepper planting and serve as alternative hosts for *A. eugenii* when pepper is scarce. Occasionally, *A. eugenii* also feeds on *Solanum melongena*, which grows around pepper plants. In Florida, USA, *A. eugenii* can thrive on Solanum americanum for up to 7 months. Therefore, promptly clearing *Solanum* weeds around cultivated land before planting chili peppers is necessary to prevent the spread and dispersal of *A. eugenii* populations [1].

The emergence of greenhouses has overcome the limitations of time and space for pepper cultivation. With the rapid development of global trade and the transportation industry for peppers, coupled with its history of expansion, the trend of *A. eugenii* spreading globally is bound to increase [9]. Consequently, a broader range of regions may be at risk than is currently projected. In future studies, the comprehensive effects of multiple environmental factors on the potential distribution of target species will be investigated, thereby providing more robust predictions.

## 5. Conclusions

We concluded that the suitable areas predicted by the ensemble model for *A. eugenii* were mainly distributed in the Midwestern and Southern United States, Central America, the La Plata Plain, parts of the Brazilian Plateau, the Mediterranean and Black Sea coasts, sub-Saharan Africa, Northern and Southern China, Southern India, Indo-China Peninsula, and coastal area in Eastern Australia. Temperature variables are important variables affecting *A. eugenii* global distribution. Under future climate scenarios, the suitability area in North America migrates northward, many areas of the Brazilian plateau become less suitable, Europe becomes more suitable and the suitability area expands to the northeast, China will become more suitable, and India and the Indo-China Peninsula become less suitable. The projections for potential distributions provide a theoretical basis for quarantine and control efforts to manage this pest, as well as provide substantial guidance for studies of the effects of climate change on other major agricultural pests.

## Figures and Tables

**Table 1:** CLIMEX parameter values for *A. eugenii*.

CLIMEX Parameter	Temperate	Semi-Arid	Final Parameter Value
Temperature requirements			
DV0—Lower temperature threshold (°C)	8	10	9.6
DV1—Lower optimum temperature (°C)	18	20	28
DV2—Upper optimum temperature (°C)	24	32	31
DV3—Upper temperature threshold (°C)	28	38	33
PDD—Degree-days per generation (°C days)	600	0	256.4
Soil moisture			
SM0—Lower soil moisture threshold	0.25	0.1	0.1
SM1—Lower optimal soil moisture	0.8	0.2	0.7
SM2—Upper optimal soil moisture	1.5	0.25	0.85
SM3—Upper soil moisture threshold	2.5	0.3	1.5
Cold stress			
TTCS—Cold stress temperature threshold (°C)	0	0	−10
THCS—Cold stress temperature rate (week^−1^)	0	0	−0.01
Heat stress			
TTHS—Heat stress temperature threshold (°C)	30	39	41.7
THHS—Heat stress temperature rate (week^−1^)	0.005	0.002	0.005
Dry stress			
SMDS—Dry stress threshold	0.2	0.05	0.1
HDS—Dry stress rate (week^−1^)	−0.005	−0.005	−0.005
Wet stress			
SMWS—Wet stress threshold	2.5	0.4	2
HWS—Wet stress rate (week^−1^)	0.002	0.01	0.001

**Table 2:** Evaluation indicators for the prediction accuracy of *A. eugenii*, *C. annuum*, and *C. frutescens* by RF.

Target Species	Climate Scenario	Period	AUC *	Kappa *	TSS *
*A. eugenii*	History	1970–2000	0.9259	0.8519	0.8710
	SSP370	2081–2100	0.8113	0.623	0.6257
	SSP585	2081–2100	0.9057	0.8117	0.8204
*C. annuum*	History	1970–2000	0.8521	0.7042	0.7189
	SSP370	2081–2100	0.8575	0.7150	0.7367
	SSP585	2081–2100	0.8654	0.7308	0.7512
*C. frutescens*	History	1970–2000	0.8566	0.7133	0.7282
	SSP370	2081–2100	0.8689	0.7380	0.7575
	SSP585	2081–2100	0.8743	0.7487	0.7695

* AUC, kappa, and TSS are 3 indicators used to evaluate model accuracy. Threshold values for discrimination abilities were AUC ≥ 0.85, kappa ≥ 0.6, and TSS ≥ 0.6.

## Data Availability

The raw data supporting the conclusions of this article will be made available by the authors on request.

## Supplementary Materials

The following supporting information can be downloaded at: https://www.mdpi.com/article/10.3390/insects16080803/s1. Table S1: Global occurrence coordinates of *A. eugenii*; Table S2: Global presence coordinates of *C. annuum*; Table S3: Global presence coordinates of *C. frutescens*.

## Abbreviations

The following abbreviations are used in this manuscript:

*A. eugenii*	*Anthonomus eugenii*
*C. annuum*	*Capsicum annuum*
*C. frutescens*	*Capsicum frutescens*
RF	Random Forests

## References

[1] Huang H., Zhou L., Lin Q., Wang Y., Wu P. (2023). Be alert to the invasion of devastating pest pepper weevil *Anthonomus eugenii* into China. J. Plant Prot..

[2] Clausen C.P., Clausen C.P. (1978). Curculionidae. Introduced Parasites and Predators of Arthropod Pests and Weeds: A World Review.

[3] Speranza S., Colonnelli E., Garonna A.P., Laudonia S. (2014). First Record of *Anthonomus eugenii* (Coleoptera: Curculionidae) in Italy. Fl. Entomol..

[4] Labbé R.M., Hilker R., Gagnier D., McCreary C., Gibson G.A.P., Fernández-Triana J., Mason P.G., Gariepy T.D. (2018). Natural enemies of *Anthonomus eugenii* (Coleoptera: Curculionidae) in Canada. Can. Entomol..

[5] Fernández D.C. (2021). An Integrated Approach to Understanding *Anthonomus eugenii* Cano (Coleoptera: Curculionidae): An Exotic Pest of Greenhouse and Field Pepper Crops. Ph.D. Thesis.

[6] Seal D.R., Martin C.G. (2016). Pepper Weevil (Coleoptera: Curculionidae) Preferences for Specific Pepper Cultivars, Plant Parts, Fruit Colors, Fruit Sizes, and Timing. Insects.

[7] Rodríguez-Leyva E., Stansly P.A., Schuster D.J., Bravo-Mosqueda E. (2007). Diversity and distribution of parasitoids of *Anthonomus eugenii* (Coleoptera: Curculionidae) from Mexico and prospects for biological control. Fl. Entomol..

[8] Rodriguez-Del-Bosque L.A., Reyes-Rosas M.A. (2003). Damage, survival, and parasitism of *Anthonomus eugenii* (Coleoptera: Curculionidae) on piquin pepper in northern Mexico. Southwest. Entomol..

[9] Fernández D.C., VanLaerhoven S.L., McCreary C., Labbé R.M. (2020). An Overview of the Pepper Weevil (Coleoptera: Curculionidae) as a Pest of Greenhouse Peppers. J. Integr. Pest. Manag..

[10] Addesso K.M., Stansly P.A., Kostyk B.C., McAuslane H.J. (2014). Organic Treatments for Control of Pepper Weevil (Coleoptera: Curculionidae). Fl. Entomol..

[11] Toapanta M.A. (2001). Population ecology, life history, and biological control of the pepper weevil, *Anthonomus eugenii* Cano (Coleoptera: Curculionidae). Ph.D. Thesis.

[12] Wu P., Haseeb M., Diedrick W., Ouyang H., Zhang R., Kanga L.H.B., Legaspi J.C. (2019). Influence of plant direction, layer, and spacing on the infestation levels of *Anthonomus eugenii* (Coleoptera: Curculionidae) in open Jalapeño pepper fields in North Florida. Fl. Entomol..

[13] (2014). IPCC Climate Change 2014: Synthesis Report. Contribution of Working Groups I, II and III to the Fifth Assessment Report of the Intergovernmental Panel on Climate Change.

[14] Saddam B., Wei C. (2025). Impact of climate change on the potential distributions of two cicada species, *Platypleura octoguttata* and *Lemuriana apicalis* (Hemiptera: Cicadidae), in India and their conservation implications. Eur. J. Entomol..

[15] Parmesan C. (2006). Ecological and Evolutionary Responses to Recent Climate Change. Annu. Rev. Ecol. Evol. Syst..

[16] Warren M.S., Hill J.K., Thomas J.A., Asher J., Fox R., Huntley B., Roy D.B., Telfer M.G., Jeffcoate S., Harding P. (2001). Rapid responses of British butterflies to opposing forces of climate and habitat change. Nature.

[17] Bentz B.J., Régnière J., Fettig C.J., Hansen E.M., Hayes J.L., Hicke J.A., Kelsey R.G., Negrón J.F., Seybold S.J. (2010). Climate Change and Bark Beetles of the Western United States and Canada: Direct and Indirect Effects. BioScience.

[18] Kraemer M.U., Sinka M.E., Duda K.A., Mylne A.Q., Shearer F.M., Barker C.M., Moore C.G., Carvalho R.G., Coelho G.E., Van Bortel W. (2015). The global distribution of the arbovirus vectors *Aedes aegypti* and *Ae. albopictus*. Elife.

[19] Deutsch C.A., Tewksbury J.J., Huey R.B., Sheldon K.S., Ghalambor C.K., Haak D.C., Martin P.R. (2008). Impacts of climate warming on terrestrial ectotherms across latitude. Proc. Natl. Acad. Sci. USA.

[20] Ning H., Dai L., Fu D., Liu B., Wang H., Chen H. (2019). Factors Influencing the Geographical Distribution of *Dendroctonus armandi* (Coleoptera: Curculionidae: Scolytidae) in China. Forests.

[21] Azrag A.G., Mohamed S.A., Ndlela S., Ekesi S. (2022). Predicting the habitat suitability of the invasive white mango scale, Aulacaspis tubercularis; Newstead, 1906 (Hemiptera: Diaspididae) using bioclimatic variables. Pest. Manag. Sci..

[22] Xie L., Wu X., Li X., Chen M., Zhang N., Zong S., Yan Y. (2024). Impacts of climate change and host plant availability on the potential distribution of *Bradysia odoriphaga* (Diptera: Sciaridae) in China. Pest. Manag. Sci..

[23] Tourinho L., Vale M.M. (2023). Choosing among correlative, mechanistic, and hybrid models of species’ niche and distribution. Integr. Zool..

[24] Elith J., Kearney M., Phillips S. (2010). The art of modelling range-shifting species. Methods Ecol. Evol..

[25] Zhang H., Zhang X., Zhang G., Sun X., Chen S., Huang L. (2024). Assessing the quality ecology of endemic tree species in China based on machine learning models and UPLC methods: The example of *Eucommia ulmoides* Oliv. J. Clean. Prod..

[26] Kriticos D.J., Maywald G.F., Yonow T., Zurcher E.J., Herrmann N.I., Sutherst R.W. (2015). CLIMEX Version 4: Exploring the Effects of Climate on Plants, Animals and Diseases.

[27] Xiao K., Ling L., Deng R., Huang B., Cao Y., Wu Q., Ning H., Chen H. (2024). Projecting the Potential Global Distribution of Sweetgum Inscriber, *Acanthotomicus suncei* (Coleoptera: Curculionidae: Scolytinae) Concerning the Host *Liquidambar styraciflua* Under Climate Change Scenarios. Insects.

[28] Yoon S., Lee W.H. (2023). Assessing potential European areas of Pierce’s disease mediated by insect vectors by using spatial ensemble model. Front. Plant Sci..

[29] Ullah F., Zhang Y., Gul H., Hafeez M., Desneux N., Qin Y. (2023). Potential economic impact of *Bactrocera dorsalis* on Chinese citrus based on simulated geographical distribution with MaxEnt and CLIMEX models. Entomol. Gen..

[30] GBIF (2024). GBIF.org GBIF Occurrence Download.

[31] GBIF (2024). GBIF.org GBIF Occurrence Download.

[32] GBIF (2024). GBIF.org GBIF Occurrence Download.

[33] Kriticos D.J., Webber B.L., Leriche A., Ota N., Macadam I., Bathols J., Scott J.K. (2012). CliMond: Global high-resolution historical and future scenario climate surfaces for bioclimatic modelling. Methods Ecol. Evol..

[34] Nakicenovic N., Alcamo J., Davis G., Vries B.d., Fenhann J., Gaffin S., Gregory K., Grubler A., Jung T.Y., Kram T. (2000). Special Report on Emissions Scenarios.

[35] Fick S.E., Hijmans R.J. (2017). WorldClim 2: New 1-km spatial resolution climate surfaces for global land areas. Int. J. Climatol..

[36] O’Neill B.C., Kriegler E., Ebi K.L., Kemp-Benedict E., Riahi K., Rothman D.S., van Ruijven B.J., van Vuuren D.P., Birkmann J., Kok K. (2017). The roads ahead: Narratives for shared socioeconomic pathways describing world futures in the 21st century. Glob. Environ. Change.

[37] Thuiller W., Georges D., Gueguen M., Engler R., Breiner F., Lafourcade B. Biomod2: Ensemble Platform for Species Distribution Modeling. https://CRAN.R-project.org/package=biomod2.

[38] R Core Team (2025). R: A Language and Environment for Statistical Computing.

[39] Chen X., Xiao K., Deng R., Wu L., Cui L., Ning H., Ai X., Chen H. (2024). Projecting the future redistribution of *Pinus koraiensis* (Pinaceae: Pinoideae: Pinus) in China using machine learning. Front. For. Glob. Chang..

[40] Fielding A.H., Bell J.F. (1997). A review of methods for the assessment of prediction errors in conservation presence/absence models. Environ. Conserv..

[41] McHugh M.L. (2012). Interrater reliability: The kappa statistic. Biochem. Med..

[42] Allouche O., Tsoar A., Kadmon R. (2006). Assessing the accuracy of species distribution models: Prevalence, kappa and the true skill statistic (TSS). J. Appl. Ecol..

[43] Hou C., Xie Y., Zhang Z. (2022). An improved convolutional neural network based indoor localization by using Jenks natural breaks algorithm. China Commun..

[44] Sutherst R.W., Maywald G.F. (1985). A computerised system for matching climates in ecology. Agric. Ecosyst. Environ..

[45] Ge X., He S., Zhu C., Wang T., Xu Z., Zong S. (2019). Projecting the current and future potential global distribution of *Hyphantria cunea* (Lepidoptera: Arctiidae) using CLIMEX. Pest. Manag. Sci..

[46] Toapanta M.A., Schuster D.J., Stansly P.A. (2005). Development and life history of *Anthonomus eugenii* (Coleoptera:Curculionidae) at constant temperatures. Environ. Entomol..

[47] Rossini L., Contarini M., Severini M., Talano D., Speranza S. (2020). A modelling approach to describe the *Anthonomus eugenii* (Coleoptera: Curculionidae) life cycle in plant protection: A priori and a posteriori analysis. Fl. Entomol..

[48] Zhu J.j., Peng Q., Liang Y.l., Wu X., Hao W.l. (2012). Leaf Gas Exchange, Chlorophyll Fluorescence, and Fruit Yield in Hot Pepper (*Capsicum anmuum* L.) Grown Under Different Shade and Soil Moisture During the Fruit Growth Stage. J. Integr. Agric..

[49] Zhu Y.F., Tan X.M., Qi F.J., Teng Z.W., Fan Y.J., Shang M.Q., Lu Z.Z., Wan F.H., Zhou H.X. (2022). The host shift of *Bactrocera dorsalis*: Early warning of the risk of damage to the fruit industry in northern China. Entomol. Gen..

[50] Xian X., Zhao H., Wang R., Huang H., Chen B., Zhang G., Liu W., Wan F. (2023). Climate change has increased the global threats posed by three ragweeds (*Ambrosia* L.) in the Anthropocene. Sci. Total Environ..

[51] Hulme P.E. (2021). Unwelcome exchange: International trade as a direct and indirect driver of biological invasions worldwide. One Earth.

[52] Warren D.L., Matzke N.J., Cardillo M., Baumgartner J.B., Beaumont L.J., Turelli M., Glor R.E., Huron N.A., Simoes M., Iglesias T.L. (2021). ENMTools 1.0: An R package for comparative ecological biogeography. Ecography.

[53] Trenberth K.E. (1990). Recent Observed Interdecadal Climate Changes in the Northern Hemisphere. Bull. Amer. Meteorol. Soc..

[54] Pysek P., Hulme P.E., Simberloff D., Bacher S., Blackburn T.M., Carlton J.T., Dawson W., Essl F., Foxcroft L.C., Genovesi P. (2020). Scientists’ warning on invasive alien species. Biol. Rev..

[55] Follett P.A., Neven L.G. (2006). Current trends in quarantine entomology. Annu. Rev. Entomol..

[56] Haack R.A., Herard F., Sun J., Turgeon J.J. (2010). Managing Invasive Populations of Asian Longhorned Beetle and Citrus Longhorned Beetle: A Worldwide Perspective. Annu. Rev. Entomol..

[57] Labbe R.M., Gagnier D., Rizzato R., Tracey A., McCreary C. (2020). Assessing New Tools for Management of the Pepper Weevil (Coleoptera: Curculionidae) in Greenhouse and Field Pepper Crops. J. Econ. Entomol..

[58] Wiens J.J., Graham C.H. (2005). Niche conservatism: Integrating evolution, ecology, and conservation biology. Annu. Rev. Ecol. Evol. Syst..

[59] Zhou Y., Guo S., Wang T., Zong S., Ge X. (2024). Modeling the pest-pathogen threats in a warming world for the red turpentine beetle (*Dendroctonus valens*) and its symbiotic fungus (*Leptographium procerum*). Pest. Manag. Sci..

[60] Newbold T. (2010). Applications and limitations of museum data for conservation and ecology, with particular attention to species distribution models. Prog. Phys. Geogr..

[61] Guisan A., Thuiller W. (2005). Predicting species distribution: Offering more than simple habitat models. Ecol. Lett..

[62] Gioria M., Osborne B.A. (2014). Resource competition in plant invasions: Emerging patterns and research needs. Front. Plant Sci..

[63] Liu C., Wolter C., Xian W., Jeschke J.M. (2020). Species distribution models have limited spatial transferability for invasive species. Ecol. Lett..

[64] Hijmans R.J. (2012). Cross-validation of species distribution models: Removing spatial sorting bias and calibration with a null model. Ecology.

[65] Helmstetter N.A., Conway C.J., Stevens B.S., Goldberg A.R. (2021). Balancing transferability and complexity of species distribution models for rare species conservation. Divers. Distrib..

[66] Qiao H., Feng X., Escobar L.E., Peterson A.T., Soberon J., Zhu G., Papes M. (2019). An evaluation of transferability of ecological niche models. Ecography.

[67] Rousseau J.S., Betts M.G. (2022). Factors influencing transferability in species distribution models. Ecography.

